# Surgical planning of osteotomies around the knee differs between preoperative standing and supine radiographs in nearly half of cases

**DOI:** 10.1186/s12891-022-05461-z

**Published:** 2022-05-26

**Authors:** Shuntaro Nejima, Ken Kumagai, Shunsuke Yamada, Masaichi Sotozawa, Dan Kumagai, Hironori Yamane, Yutaka Inaba

**Affiliations:** Department of Orthopaedic Surgery, Yokohama City University School of Medicine, 3-9 Fukuura, Kanazawa-ku, 236-0004 Yokohama, Japan

**Keywords:** Open-wedge high tibial osteotomy, Closed-wedge high tibial osteotomy, Double-level osteotomy, Distal femoral osteotomy, Osteotomies around the knee

## Abstract

**Background:**

To evaluate the difference in surgical planning of osteotomies around the knee between preoperative standing and supine radiographs and to identify risk factors for discrepancies in surgical planning.

**Methods:**

This study included 117 knees of 100 patients who underwent osteotomies around the knee for knee osteoarthritis with genu varum. Surgical planning was performed so that the target point of the postoperative weight-bearing line (WBL) ratio was 62.5% in preoperative standing and supine radiographs. If the opening gap would be > 13 mm in open-wedge high tibial osteotomy (OWHTO), closed-wedge HTO (CWHTO) was planned. If the postoperative mMPTA would be > 95° in isolated HTO, double-level osteotomy (DLO) was planned. In DLO, lateral closed-wedge distal femoral osteotomy was performed so that the postoperative mechanical lateral distal femoral angle (mLDFA) was 85°, and any residual varus deformity was corrected with HTO.

**Results:**

Surgical planning differed between standing and supine radiographs in 43.6% of cases. In all knees for which surgical planning differed between standing and supine radiographs, a more invasive type of osteotomy was suggested by standing radiographs than by supine radiographs. The risk factors for discrepancies in surgical planning were a lower WBL ratio in standing radiographs and a lower joint line convergence angle in supine radiographs.

**Conclusions:**

Surgical planning of DLO, CWHTO and OWHTO, in standing radiographs differed from that in supine radiographs in nearly half of the cases. Surgical planning based on standing radiographs leads to more invasive surgical procedures compared to supine radiographs.

## Background

Osteotomy around the knee is a well-established procedure for medial knee osteoarthritis (OA) with varus alignment, and several types of osteotomies have been introduced. Open-wedge high tibial osteotomy (OWHTO) has become increasingly common as a result of improved surgical techniques [1, 2]. Although the overall clinical results of OWHTO were good [3], some complications were reported [4–6]. Increased opening width was a risk factor for degeneration of the patellofemoral joint and delayed bone union after OWHTO [7, 8]. Closed-wedge high tibial osteotomy (CWHTO) could be an alternative surgical procedure for such cases [9]. In addition, excessive postoperative overcorrection of the medial proximal tibial angle (MPTA) to more than 95° following isolated HTO leads to increased shear stress in the medial cartilage [10] and, consequently, inferior clinical outcomes [11, 12]. Double-level osteotomy (DLO), which includes distal femoral osteotomy (DFO) and HTO, was introduced to prevent overcorrection of the proximal tibia; good clinical results were reported with this procedure [13–18]. To prevent inferior clinical results, not only OWHTO but also DLO and CWHTO should be included as surgical options.

One of the major problems regarding osteotomies around the knee is the postoperative change in soft tissue balance. A postoperative decrease in the joint line convergence angle (JLCA) leads to overcorrection of the lower limb alignment overall [19–22]. Although a relationship between preoperative radiological parameters and postoperative changes in soft tissue balance has been reported [19–25], it seems to be difficult to accurately predict postoperative changes in soft tissue balance. To predict postoperative soft tissue balance, preoperative supine radiographs have been reported to be useful because the preoperative JLCA in supine radiographs is similar to the postoperative JLCA in standing radiographs [26, 27].

There is a possibility that the surgical planning of osteotomies around the knee, including OWHTO, CWHTO and DLO, will differ depending whether the plans are based on preoperative standing or supine radiographs. Surgical planning based on standing radiographs may lead to more invasive surgical procedures than necessary, but this possibility has not been evaluated. Thus, the purpose of this retrospective study was to evaluate the difference in surgical planning of osteotomies around the knee between preoperative standing and supine radiographs. In addition, this study aimed to identify risk factors for discrepancies in surgical planning between preoperative standing and supine radiographs. It was hypothesized that a considerable number of knees would show discrepancies in surgical planning and that a large difference in JLCA between standing and supine radiographs would be a risk factor for discrepancies in surgical planning.

## Methods

 This study was approved by the institutional review board of Yokohama City University (F211100004), and written informed consent was obtained from each patient. A total of 154 patients (189 knees) who underwent osteotomies around the knee for medial knee OA with varus alignment between August 2016 and July 2021 were enrolled. Patients with a concomitant procedure, a history of surgical procedures on the lower limbs or a lack of long-leg radiographs in the supine position were excluded. Thus, 100 patients (117 knees) met the inclusion criteria for this study.

### Measurements of joint orientation angles on radiographs

For preoperative surgical planning, anterior–posterior long-leg radiographs were obtained with the knee in full extension in the one-leg standing and supine positions. The patella was positioned centrally between the femoral condyles. The hip-knee-ankle angle (HKA), weight-bearing line (WBL) ratio, and JLCA were analysed in standing and supine radiographs, and the mechanical lateral distal femoral angle (mLDFA) and mechanical medial proximal tibial angle (mMPTA) were analysed in standing radiographs. The analyses of radiological parameters and the surgical planning were performed using Fujifilm OP-A software (Fujifilm Co. Ltd., Tokyo, Japan). The HKA was defined as the angle between the mechanical axes of the femur and tibia, with a negative value representing varus alignment. The WBL ratio was defined as the point at which the mechanical axis of the lower limb passed through the tibial plateau. The medial and lateral margins of the tibial plateau were defined as 0 and 100%, respectively. The mLDFA was defined as the lateral angle between the mechanical axis of the femur and the articular surface of the distal femur. The JLCA was defined as the angle between the articular surfaces of the distal femur and proximal tibia. The JLCA was presented as positive if there was an acute angle on the medial side. The mMPTA was defined as the medial angle between the mechanical axis of the tibia and the articular surface of the proximal tibia. Good intra- and inter-observer reliability values have been reported for these joint orientation angles [28]. The differences in the HKA, WBL ratio and JLCA between standing and supine radiographs (parameters in supine position – parameters in standing position) were defined as ΔHKA, ΔWBL ratio and ΔJLCA, respectively.

### Surgical planning of osteotomies around the knee

**Fig. 1:**
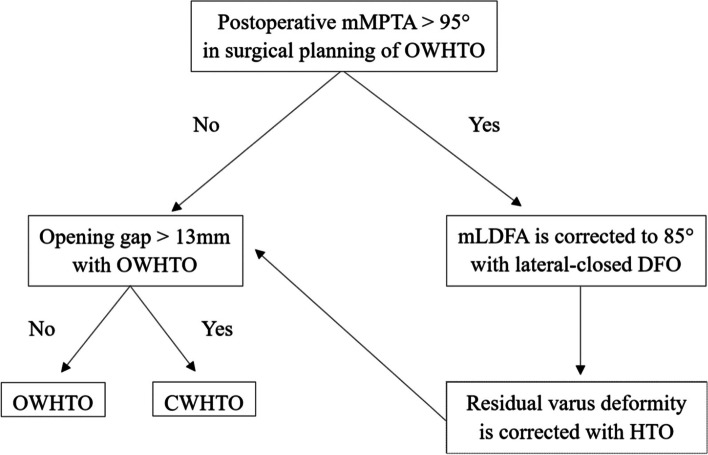
Surgical planning was performed so that the target point of the postoperative weight-bearing line ratio was 62.5%. First, open-wedge high tibial osteotomy (HTO) was planned. If the opening gap was larger than 13 mm, closed-wedge HTO was planned. If the postoperative mechanical medial proximal tibial angle was over 95° with isolated HTO, double-level osteotomy (DLO) was planned. In DLO, lateral closed-wedge distal femoral osteotomy was performed so that the postoperative mechanical lateral distal femoral angle was 85°, and any residual varus deformity was corrected with HTO

Surgical planning was performed so that the target point of the postoperative WBL ratio was 62.5% in standing and supine radiographs. First, OWHTO was planned. A transverse cut was made 35 mm below the medial joint to the safe zone [29]. A lateral hinge of 5 mm was left. If the opening gap was larger than 13 mm, CWHTO was planned because an opening gap larger than 13 mm was a risk factor for delayed bone union and progression of patellofemoral cartilage damage [7, 8]. CWHTO was planned in a hybrid style [9, 30, 31]. A transverse cut was made 40 mm below the lateral tibial plateau to the inflection point of the medial tibial cortex. The hinge point was set to divide the transverse cut line by 3 to 1. If the postoperative mMPTA was over 95° with isolated HTO, DLO was planned [16, 17]. In DLO, lateral closed-wedge DFO was performed so that the postoperative mLDFA was 85° [16, 32], and any residual varus deformity was corrected with HTO (Fig. 1). Knees with discrepancies in surgical planning between standing and supine radiographs were defined as the discrepancy group, and all other knees were classified in the matched group. In addition, knees with postoperative mMPTA values ≤ 95° and > 95°, respectively, were defined as the correctable group and the uncorrectable group.

### Statistical analysis

Data are presented as the means and standard deviations. The results were analysed using IBM SPSS for Windows, version 27.0 (IBM Corporation, Armonk, NY, USA). Patient characteristics and preoperative radiological parameters were compared between the discrepancy and matched groups using the chi-squared test and the unpaired t test. Preoperative HKA, WBL ratio and JLCA values were compared between standing and supine radiographs using the paired t test. Binary logistic regression analysis with forward selection was performed to identify risk factors associated with the discrepancy group. In addition, the ratio of uncorrectable knees was compared between standing and supine radiographs using the McNemar test. A *P* value less than 0.05 was considered significant. A post hoc power analysis was performed on the comparisons using an unpaired t test (significance level = 0.05, effect size = 0.33 from supine JLCA, sample size = 51 and 66) in G*Power version 3.1.9.2 (Heinrich-Heine-Universität, Düsseldorf, Germany) and resulted in a power of 0.42.

## Results

The patients’ demographic data are presented in Table 1. Of the 117 knees, 51 knees (43.6%) were classified into the discrepancy group. Kellgren-Laurence grades were more severe in the discrepancy group than in the matched group. The mMPTA, both the standing and supine HKA and WBL ratio, and the standing JLCA were more varus in the discrepancy group than in the matched group. In addition, ΔHKA, ΔWBL ratio and ΔJLCA were larger in the discrepancy group than in the matched group. On preoperative standing radiographs, the HKA, WBL ratio and JLCA were more varus than on supine radiographs (Table 2).

**Table 1:** Patients’ demographic and preoperative radiological data

Variables	Discrepancy group (*n* = 51)	Matched group (*n* = 66)	*P* value
Age, y	64.6 ± 8.4 (45–83)	66.1 ± 7.4 (47–79)	n.s.
Body mass index, kg/m^2^	26.4 ± 3.7 (20.0–40.2)	26.8 ± 3.8 (19.3–36.6)	n.s.
Side, left/right	28/23	30/36	n.s.
Gender, male/female	18/33	23/43	n.s.
Kellgren-Laurence grade 1/2/3/4	0/0/3/48	0/6/22/38	< 0.001
mLDFA, °	88.0 ± 1.5 (83.4–92.1)	87.5 ± 2.6 (82.2–93.6)	n.s.
mMPTA, °	82.9 ± 2.5 (75.4–90.1)	84.8 ± 2.1 (79.1–89.4)	< 0.001
Standing HKA, °	−11.0 ± 3.2 (− 20.8 – −3.5)	−7.4 ± 3.7 (− 19.4 – −0.1)	< 0.001
Supine HKA, °	−8.2 ± 2.6 (− 16.5 – −2.2)	−5.8 ± 3.0 (− 14.7 – −0.6)	< 0.001
ΔHKA, °	2.8 ± 1.8 (− 3.7–6.5)	1.6 ± 1.2 (− 1.0–4.7)	< 0.001
Standing WBL ratio, %	−2.9 ± 14.7 (− 36.9–32.6)	13.7 ± 16.0 (− 38.7–44.0)	< 0.001
Supine WBL ratio, %	11.1 ± 11.3 (− 20.1–36.1)	21.8 ± 12.6 (− 13.0–45.9)	< 0.001
ΔWBL ratio, %	14.0 ± 8.3 (− 11.1–32.8)	8.1 ± 5.4 (− 0.3–25.7)	< 0.001
Standing JLCA, °	5.9 ± 2.1 (2.2–9.8)	4.8 ± 2.3 (0.7–10.8)	< 0.05
Supine JLCA, °	2.0 ± 1.9 (− 1.7–6.6)	2.7 ± 2.3 (− 2.1–9.9)	n.s.
ΔJLCA, °	−3.9 ± 2.1 (− 10.5 – −0.3)	−2.1 ± 1.8 (− 6.2–1.7)	< 0.001

Data are presented as means ± standard deviation with the range in parentheses. *mLDFA* mechanical lateral distal femoral angle, *mMPTA* mechanical medial proximal tibial angle, *HKA* hip-knee-ankle angle, *WBL* weight-bearing line, *JLCA* joint line convergence angle

**Table 2:** Preoperative HKA, WBL ratio and JLCA in standing and supine radiographs

Variables	Standing radiographs	Supine radiographs	*P* value
HKA, °	−9.0 ± 3.9 (− 20.8 – −0.1)	−6.8 ± 3.1 (− 16.5 – −0.6)	< 0.001
WBL ratio, %	6.4 ± 17.5 (− 38.7–44.0)	17.1 ± 13.1 (− 20.1–45.9)	< 0.001
JLCA, °	5.3 ± 2.3 (0.7–10.8)	2.4 ± 2.2 (− 2.1–9.9)	< 0.001

Data are presented as means ± standard deviation with the range in parentheses. *HKA* hip-knee-ankle angle, *WBL* weight-bearing line, *JLCA* joint line convergence angle

The types of osteotomy selected based on standing and supine radiographs are shown in Table 3. DLO was selected based on standing radiographs in 71 knees (60.7%) and based on supine radiographs in 37 knees (31.6%). Among the 71 knees indicated for DLO based on standing radiographs, 34 knees (47.9%) were indicated for HTO or DFO based on supine radiographs. On the other hand, among the 37 knees indicated for DLO based on supine radiographs, no knees were indicated for HTO or DFO based on standing radiographs.

**Table 3:** The surgical planning in standing and supine radiographs (*n* = 117)

		The surgical planning in supine radiographs					
		DFO + CWHTO	DFO + OWHTO	CWHTO	OWHTO	DFO	Total
The surgical planning in standing radiographs	DFO + CWHTO	3 (2.6%)	10 (8.5%)	11 (9.4%)	3 (2.6%)	0	27 (23.1%)
	DFO + OWHTO	0	24 (20.5%)	7 (6.0%)	12 (10.3%)	1 (0.9%)	44 (37.6%)
	CWHTO	0	0	2 (1.7%)	7 (6.0%)	0	9 (7.7%)
	OWHTO	0	0	0	37 (31.6%)	0	37 (31.6%)
	DFO	0	0	0	0	0	0
	Total	3 (2.6%)	34 (29.1%)	20 (17.1%)	59 (50.4%)	1 (0.9%)	117

*DFO* distal femoral osteotomy, *OWHTO* open-wedge high tibial osteotomy, *CWHTO* closed-wedge high tibial osteotomy

Binary logistic regression analysis with forward selection showed that a lower preoperative standing WBL ratio and supine JLCA were independent predictors of membership in the discrepancy group (Table 4). In contrast, the analysis did not show age, body mass index, gender, mLDFA, mMPTA, standing HKA, supine HKA, ΔHKA, supine WBL ratio, ΔWBL ratio, standing JLCA or ΔJLCA to be a risk factor.

**Table 4:** Binary logistic regression analysis of risk factors for mismatched group

Variables	Coefficient	Standard error	Wald	Odds ratio (95% CI)	*P* value
Standing WBL ratio	−0.088	0.018	24.424	0.916 (0.884–0.948)	< 0.001
Supine JLCA	−0.377	0.116	10.486	0.686 (0.546–0.862)	0.001

*WBL* weight-bearing line, *JLCA* joint line convergence angle

The proportion of knees with postoperative mMPTA > 95° in supine radiographs (3/117 knees, 2.6%) was lower than the proportion in standing radiographs (34/117 knees, 29.1%) (*P* < 0.001).

## Discussion

The most important finding of this study was that surgical planning of procedures including OWHTO, CWHTO and DLO differed between standing and supine radiographs in 43.6% of legs that underwent osteotomies around the knee for medial knee OA with varus alignment.

The present study indicated that surgical planning of osteotomies around the knee using standing radiographs could lead to a more invasive surgical procedure than necessary. Generally, surgical planning of osteotomies around the knee is performed using standing radiographs [3]. However, postoperative changes in soft tissue balance lead to overcorrection of lower limb alignment, and the necessity of adjusting the amount of correction in surgical planning of HTO has been reported [19–22]. The utility of preoperative supine radiographs as a means to predict postoperative soft tissue balance in standing radiographs has also been described in the literature [26, 27]. Ogata et al. [26] reported that in CWHTO, the preoperative JLCA in the supine position was similar to the postoperative JLCA in the standing position. In addition, the mean value of the preoperative JLCA in the supine position did not differ from that of the postoperative JLCA in the standing position in OWHTO [27]. Among the 43.6% of knees in which surgical planning differed between standing and supine radiographs in the present study, the surgical procedures planned from standing radiographs seemed to be more invasive than those planned from supine radiographs. Moreover, the present study revealed that in knees indicated for DLO based on standing radiographs, nearly half of cases were indicated for HTO or DFO based on supine radiographs. In CWHTO, a fibular osteotomy is needed, and adjusting alignment during surgery is more difficult than in OWHTO. DLO has additional risks such as hinge fracture [33] and popliteal artery injury during DFO [16, 34]. In a previous study, the amount of correction in HTO was greater when planned from standing radiographs than when planned from supine radiographs [35]. The present study indicated that more invasive surgical procedures were likely to be chosen when surgical planning was based only on standing radiographs.

In the present study, the risk factors for discrepancies in surgical planning between standing and supine radiographs were a lower WBL ratio in standing radiographs and a lower JLCA in supine radiographs. The difference in the JLCA between standing and supine radiographs was the most important preoperative factor predicting the coronal correction discrepancy after OWHTO in a previous study [23]. Considering this result, we hypothesized that ΔJLCA was a risk factor for discrepancies in surgical planning between standing and supine radiographs. Thus, our hypothesis was not supported. Although the reason was unclear, a possible explanation is that surgical planning was performed based on the WBL ratio in standing radiographs. Moreover, the change in soft tissue balance could be affected not only by JLCA but also by other factors such as coronal tibiofemoral subluxation [36, 37].

The present study also indicated that surgical planning using standing radiographs could lead to excessive overcorrection of the mMPTA. A previous study using a three-dimensional finite element model showed that joint line obliquity (JLO) of more than 5° induced excessive shear stress in the medial compartment of the knee [10]. In addition, postoperative MPTA > 95° led to inferior clinical results in OWHTO [11, 12]. DLO prevented excessive postoperative overcorrection of mMPTA and JLO and led to superior Lysholm scores compared to OWHTO [17]. The present study showed that the proportion of knees with postoperative mMPTA values > 95° was 29.1% when the surgical planning was based on standing radiographs, although DLO was included among the surgical options. Meanwhile, the proportion of knees with postoperative mMPTA values > 95° was only 2.6% when the surgical planning was based on supine radiographs. Considering that the postoperative JLCA in standing radiographs is closer to the preoperative JLCA in supine radiographs than in standing radiographs, surgical planning with standing radiographs could lead to unnecessary overcorrection of the mMPTA and inferior clinical outcomes after surgery.

Considering the results of the present and previous studies [26, 27], supine radiographs should be evaluated in surgical planning to prevent invasive surgical procedures and excessive overcorrection of the mMPTA. Shin et al. [27] reported that preoperative planning with supine radiographs was more accurate than preoperative planning with standing radiographs as a predictor of postoperative whole lower limb alignment in OWHTO. In their study, the mean value of the postoperative WBL ratio in standing radiographs was 61.95% when surgical planning of OWHTO was performed using supine radiographs, and the target point of the WBL ratio was set to 62.5%. Although the mean value of the postoperative WBL ratio was close to 62.5%, the postoperative WBL ratio seemed to range from approximately 50–75% in their study [27]. This result indicates that although the surgical planning was performed using supine radiographs, the postoperative alignment was not completely consistent with preoperative planning. However, the range of postoperative WBL ratios mentioned above was considered acceptable in previous studies. Hohloch et al. [38] reported that knees with a WBL ratio of 50–55% achieved better Lysholm scores and Knee Injury and Osteoarthritis Outcome Score pain scores than knees with a ratio of 60–65% in patients who underwent OWHTO. Moreover, Lee et al. [39] reported that the Western Ontario and McMaster Universities Osteoarthritis index and the International Knee Documentation Committee subjective score were not different between knees aiming at lateral tibial supine (the mean WBL ratio was 57.4 ± 1.6%) and 62.5%. El-Azab et al. [40] reported that overcorrection was not associated with poorer clinical outcomes than accurate correction. Thus, surgical planning aiming at a 62.5% WBL ratio with supine radiographs can be effective for preventing unnecessarily invasive surgical procedures and excessive overcorrection of the mMPTA and lead to acceptable clinical outcomes, although the range of postoperative WBL ratios is relatively wide.

There were a few limitations in this study. First, this study was based on radiological surgical simulation without clinical outcomes in patients who underwent osteotomies around the knee. To confirm the superiority of preoperative surgical planning with supine radiographs compared to standing radiographs, further studies are needed in the future. Second, the sample size of this study was relatively small. Third, there are various surgical planning algorithms for osteotomies around the knee [16, 28]. In the present study, the target point of the postoperative WBL ratio was 62.5%. In addition, CWHTO was planned when the opening gap was over 13 mm in OWHTO, and DLO was planned when the postoperative mMPTA was over 95° with isolated HTO. Further studies are needed to investigate the best algorithm for surgical planning of osteotomies around the knee. Fourth, both knees in 17 patients were analysed in the present study. Although it is unclear whether the measured values or the surgical planning on preoperative radiographs differs between bilateral lower limbs, this could bias the results in the present study.

Regarding the practical clinical implications of this study, preoperative supine radiographs should be evaluated in surgical planning of osteotomies around the knee to prevent unnecessarily invasive surgical procedures and excessive overcorrection of mMPTA.

## Conclusions

Surgical planning of procedures including DLO, CWHTO and OWHTO differed between standing and supine radiographs in nearly half of cases. Surgical planning based on standing radiographs led to more invasive surgical procedures and excessive postoperative overcorrection of the proximal tibia compared to supine radiographs.

## Data Availability

The datasets used and/or analysed during the current study available from the corresponding author on reasonable request.

## Abbreviations

OA: Osteoarthritis
OWHTO: Open-wedge high tibial osteotomy
CWHTO: Closed-wedge high tibial osteotomy
MPTA: Medial proximal tibial angle
DLO: Double level osteotomy
DFO: Distal femoral osteotomy
JLCA: Joint line convergence angle
HKA: Hip-knee-ankle angle
WBL: Weight-bearing line
mLDFA: Mechanical lateral distal femoral angle
mMPTA: Mechanical medial proximal tibial angle
JLO: Joint line obliquity
